# Response to: Comment on “Factors Associated with Recurrent Ulcers in Patients with Gastric Surgery after More Than 15 Years: A Cross-Sectional Single-Center Study”

**DOI:** 10.1155/2019/9214597

**Published:** 2019-02-24

**Authors:** Anca Negovan, Monica Pantea, Claudia Banescu, Simona Mocan

**Affiliations:** ^1^Clinical Science-Internal Medicine, University of Medicine and Pharmacy, Gheorghe Marinescu 38, Tirgu Mureș 540139, Romania; ^2^Center for Advanced Medical and Pharmaceutical Research, University of Medicine and Pharmacy, Gheorghe Marinescu 38, Tirgu Mureș, 540139 Mures, Romania; ^3^Pathological Department, Emergency County Hospital, Gheorghe Marinescu 50, Tirgu Mures, 540136 Mures, Romania

We would like to thank Dr. D. Ribaldone and Dr. R. Pellicano for their interest [1] in our article “Factors Associated with Recurrent Ulcers in Patients with Gastric Surgery after More Than 15 Years: A Cross-Sectional Single Center Study” [2]. They raised the problem of false-negative results of *Helicobacter pylori* (*H. pylori*) infection in patients with endoscopic lesions. We started the present research after several years of clinical observations in patients with gastric surgery for peptic ulcer disease. The lack of association between mucosal lesions and the most important etiologic factor for ulcer in our population, *H. pylori* infection, determined us to investigate it systematically. Anyway, the results are not very surprising as the clearance of infection occurs in time, after surgery, due to the effect of bile, the progression of atrophic gastritis, intestinal metaplasia, and the gastric pH changes [3]. In order to rule out a possible confounding factor, we excluded from the beginning the patients with a prior proton pump inhibitor (PPI) or eradication therapy for *H. pylori*, as mentioned in the Method chapter [2]. The biopsies were taken from the very upper part of the corpus, taking into consideration the progression of the topography of gastritis [4]. After standard histochemical staining and examination, when *H. pylori* infection is suggested by the presence of gastritis (active or inactive) or by extensive intestinal metaplasia, the immunohistochemical study is performed. In order to avoid the false-negative results, this approach is routinely used when no organisms are identified, but a high suspicion of infection persists, in accordance with the current recommendation [5]. Using a second test after histological examination could not be an option, as the decreased *H. pylori* load in gastric mucosa may lead to false-negative results in both the stool antigen test and the urea breath test [6, 7]. One strength of our study is the systematic approach of histologic changes in the gastric remnant. From 76 patients with gastric surgery, only 8 patients (10.5%) were *H. pylori*-positive, a low frequency in comparison with our previous reports that found 33%-40% *H. pylori*-positive patients from consecutive patients investigated on endoscopy [8, 9]. Nevertheless, the frequency of reactive gastropathy was 73% among resected patients in comparison with 34% in our previous series without gastric surgery, supporting the role of chemical aggression in gastric stump, not inflammation [8]. Endoscopic lesions were borderline correlated with low-dose aspirin consumption in our study, and only the small number of cases did not allow us to reveal the role of drug-induced lesions. Our research aimed to evaluate the influenced factors to avoid long-term complications after gastric surgery and support the diminished role of infection in the studied population.

In conclusion, in patients with long-standing gastric surgery, chemical aggression and reactive gastropathy in gastric mucosa, but not inflammatory changes due to *H. pylori* infection, play a more important role in endoscopic lesion occurrence. In our opinion, the histological examination assessing the cell morphology is the recommended approach to avoid false-negative results for *H. pylori* infection in the research settings.
